# A Descriptive Catalogue of the Anatomical Museum of the Boston Society for Medical Improvement

**Published:** 1848-05

**Authors:** 


### EDITORIAL DEPARTMENT.

*A Descriptive Catalogue of the Anatomical Museum of the Boston Society for Medical improvement.* By J. B. S. Jackson, M. D., Curator of the Museum, and Professor of Pathological Anatomy in Harvard University. Boston, Wm. D. Ticknor & Co. 1847. 8vo. pp. 352.

This is a beautifully gotten up book, creditable alike to publishers, the society under whose auspices it is issued, and the accomplished Professor whose name appears on the title page. To ourselves it is a most welcome volume, aside from the intrinsic value which it possesses for every lover of Pathological Anatomy: it reproduces objects which we once had the pleasure examining, and, at the same time, listening to the explanations and valuable comments of our friend—the able and indefatigable Curator of the Museum. Although the interest and value of the Catalogue are enhanced by this circumstance, yet, for those who have not been so fortunate as to enjoy the same privilege, the work must prove both attractive and instructive; and we conceive that its publication will be of no inconsiderable service in stimulating other associations to follow the example of the Boston Society, in collecting and preserving specimens illustrative of the various lesions of which the solid structures are the seat. Prof. Jackson, as we have been informed, is in a great measure entitled to the merit of making the Museum of the Society for mental improvement what it is—a source of scientific profit, and of just pride to his professional brethren of Boston. By extending the advantages of the collection by means of this carefully prepared descriptive catalogue, he has laid the profession of the whole country under obligations.

A detailed analysis of a work of this description is, of course, out of the question. In order, however, that the reader may form some idea of its scope, we copy his classification, which is an anatomical one, together with the number of specimens belonging to each division:

| | | | |
|---|---|---|---|
| 1. | Healthy Bones, | 78 | specimens. |
| 2. | Diseased Bones, | 227 | " |
| 3. | Soft parts about bones, | 22 | " |
| 4. | Heart and Blood-vessels, | 63 | " |
| [This department is especially rich in rare and valuable specimens.] | | | |
| 5. | Organs of Sense, | 212 | " |
| 6. | Vocal and Respiratory Organs, | 29 | " |
| 7. | Alimentary Canal, | 85 | " |
| 8. | Organs accessory to Alimentary Canal, | 55 | " |
| 9. | Urinary Organs, | 65 | " |
| 10. | Female Organs of Generation, | 38 | " |
| 11. | Male Organs of Generation, | 24 | " |
| 12. | Utero-gestation, | 45 | " |
| 13. | Monstrosities, | 124 | " |
| 14. | Parasites, | 34 | " |
| 15. | Miscellaneous, | 25 | " |
| | Total 924. | | |

The formation of a museum of Pathological Anatomy is one of the means by which members of an association are incited to attend meetings, and to co-operate for the general objects for which scientific associations are formed. In this way, indirectly, as well as directly, it accomplishes good. We are happy to learn by the following concluding paragraph of the preface of the work before us that this effect has been exerted upon the Boston society for mutual improvement:—

"In regard to the general prospects of the society they were never better than at the present time. The meetings are well attended, and well sustained, and tend to the promotion of good feeling, as well as of medical science. The cabinet, the library, and the records of nearly twenty years, faithfully kept, form a substantial basis. The same spirit that founded the society, and has thus far actuated its members, still prevails, and, it is hoped, will perpetuate its existence, and render it for many years to come deserving of the significant name it bears."

In concluding this article, we cannot forbear expressing regret that the same spirit which has actuated the Curator in the publication of this catalogue does not lead the society to extend the benefits of the society beyond the circle of its members, by permitting some of the most valuable of the "records of twenty years," to appear in the columns of a medical journal, or some other vehicle for the professional reader. We shall not refrain from quarreling with our Boston friends whenever they circumscribe their useful labors within the limits of their own city.

